# Adenosine Receptor A2B Negatively Regulates Cell Migration in Ovarian Carcinoma Cells

**DOI:** 10.3390/ijms23094585

**Published:** 2022-04-21

**Authors:** Anaí del Rocío Campos-Contreras, Adriana González-Gallardo, Mauricio Díaz-Muñoz, Francisco G. Vázquez-Cuevas

**Affiliations:** Departamento de Neurobiología Celular y Molecular, Instituto de Neurobiología, Campus UNAM-Juriquilla, Querétaro 76230, CP, Mexico; anaicampos.c@gmail.com (A.d.R.C.-C.); gallardog@unam.mx (A.G.-G.); mdiaz@comunidad.unam.mx (M.D.-M.)

**Keywords:** purinergic signaling, A2B receptor, ovarian cancer, cancer cell migration, SKOV-3 cells

## Abstract

The purinergic system is fundamental in the tumor microenvironment, since it regulates tumor cell interactions with the immune system, as well as growth and differentiation in autocrine-paracrine responses. Here, we investigated the role of the adenosine A2B receptor (A2BR) in ovarian carcinoma-derived cells’ (OCDC) properties. From public databases, we documented that high A2BR expression is associated with a better prognostic outcome in ovarian cancer patients. In vitro experiments were performed on SKOV-3 cell line to understand how A2BR regulates the carcinoma cell phenotype associated with cell migration. RT-PCR and Western blotting revealed that the *ADORA2B* transcript (coding for A2BR) and A2BR were expressed in SKOV-3 cells. Stimulation with BAY-606583, an A2BR agonist, induced ERK1/2 phosphorylation, which was abolished by the antagonist PSB-603. Pharmacological activation of A2BR reduced cell migration and actin stress fibers; in agreement, A2BR knockdown increased migration and enhanced actin stress fiber expression. Furthermore, the expression of E-cadherin, an epithelial marker, increased in BAY-606583-treated cells. Finally, cDNA microarrays revealed the pathways mediating the effects of A2BR activation on SKOV-3 cells. Our results showed that A2BR contributed to maintaining an epithelial-like phenotype in OCDC and highlighted this purinergic receptor as a potential biomarker.

## 1. Introduction

Extracellular adenosine (ADO) is originated from the ectonucleotidase-mediated hydrolysis of extracellular ATP released by tumor cells into the tumor microenvironment (TME), mainly by the CD39-CD73 pathway [1]. It is well established that ATP concentration in the TME is significantly higher than in healthy tissue [2]; moreover, in some anticancer therapies, ATP released from dying tumor cells contributes to maintaining an elevated concentration of this nucleotide in the TME [3]. Correspondingly, ADO increases in cancer conditions [4]. In the TME, ADO acts as a mediator in the interaction among host immunological cells to induce tumor-immune evasion [5,6] and displays autocrine-paracrine actions to regulate cellular processes such as proliferation, migration and differentiation in tumor cells [7,8,9,10,11]. 

ADO exerts its actions through P1 receptors, which belong to the G protein-coupled receptor (GPCR) superfamily. The P1 receptor family has four members: A1R, A2AR, A2BR and A3R. In general, A1R and A3R receptors are coupled mainly to Gi proteins, whereas A2AR and A2BR are coupled to Gs proteins [12]. In addition, A2BR regulates various effectors through different pathways; that is, mitogen-activated protein kinases p38 and ERK [13], and is also coupled to Gq proteins to promote PLC-IP_3_-Ca^2+^ pathway activity [14]. 

The present work sought to characterize the autocrine–paracrine actions mediated by A2BR in SKOV-3 OCDC. In addition, A2BR expression has been demonstrated in biopsies from hepatocellular [15], colorectal [16] and bladder urothelial [17] carcinoma. A2BR activity promoted cell proliferation in breast cancer cells [18].

An important feature of cancer cells is their ability to migrate and invade secondary organs. To dissociate from primary tumors and survive in peritoneal fluid, cells go through epithelial–mesenchymal transition (EMT), switching from an epithelial to a mesenchymal phenotype [19]. Purines are active modulators of EMT induction in cancer [20]. 

ADO/A2BR, specifically, has been demonstrated to regulate cell migration in several tissues; thus, A2BR activation increases cell migration in MDA-MB-231 breast cancer line [18]. Accordingly, pharmacological inhibition of A2BR decreases cell migration in urothelial and renal cancer cell lines [17,21]. On the other hand, in human cervical cancer cells, extracellular ADO inhibits migration and invasion [22], and in epithelial lung cancer cells, A2BR activity counteracts the TFG-β–dependent EMT induction [23]. In SKOV-3 cells, addition of apyrase (Apy), which hydrolyzes extracellular ATP generating ADO, was shown to decrease cell migration and favor an epithelial-like phenotype due to the relocation of E-cadherin to cellular junctions, probably through A2BR [24]. In the present study, we analyzed this possibility.

## 2. Results

### 2.1. ADORA2B Expression Is Related to the Surveillance Probability of Ovarian Carcinoma Patients and Is Specific to the Tumor Type

We analyzed if the expression level of the *ADORA2B* transcript (coding for A2BR) correlated with the surveillance probability of ovarian carcinoma patients using the Kaplan–Meier Plotter database (K-MPdb) [25,26]. K-MPdb contains the transcript expression level data collected from different transcriptomic studies, in correlation with the surveillance of 1435 ovarian cancer patients. 

**Figure 1:**
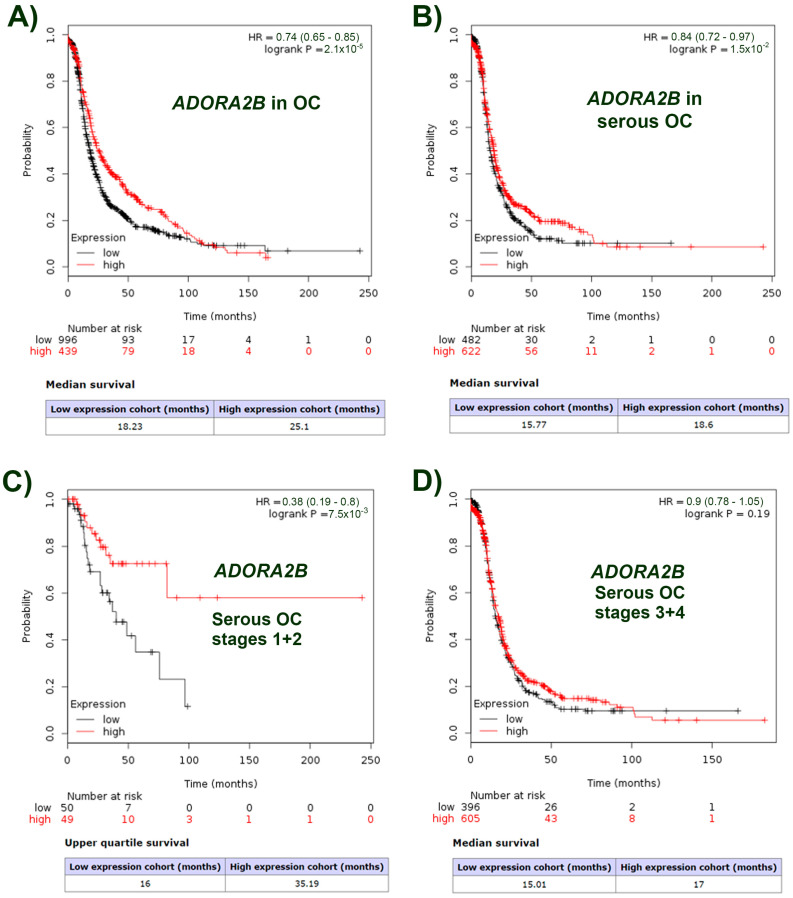
Relation between *ADORA2B* transcript (coding for A2BR) and survival time for patients with OC. Kaplan–Meier plots were constructed for the *ADORA2B* transcript using the Kaplan–Meier plotter database (KMPdb) for (**A**) Ovarian carcinoma in general carcinoma patients (996 low expression and 439 high expression); (**B**) Serous ovarian carcinoma patients (482 low expression and 622 high expression); (**C**) Serous ovarian carcinoma patients in stages 1 + 2 (50 low expression and 49 high expression) and (**D**) Serous ovarian carcinoma patients in stages 3 + 4 (396 low expression and 605 high expression).

The first analysis correlated the expression level of the *ADORA2B* transcript with the survival rate of ovarian cancer patients (996 of low expression and 439 of high expression) (Figure 1A). We observed that the survival was different between both groups of patients: the reduced expression of the transcript showed a lower probability of survival (18.23 months), whereas the higher expression showed an increased survival (25.10 months, *p* = 0.00002). Then, we made the K-MPdb analysis according to the type of carcinoma: for the endometrioid subtype, no differences were detected between 15 patients with low expression and 36 patients with high expression of *ADORA2B* (*p* = 0.19, data not shown). In contrast, for serous carcinoma, the surveillance of both cohorts (482 with low expression vs. 622 with high expression) revealed that the group with reduced *ADORA2B* had a worse prognosis than the group with high *ADORA2B* levels (15.80 months vs. 18.60 months, respectively) (*p* = 0.015) (Figure 1B). 

To know if the tumor stage in serous carcinoma was relevant for the direct relation between *ADORA2B* expression level and patient survival, K-MPdb analysis was also performed considering tumor stages (1 + 2 or 3 + 4). Interestingly, for patients in stages 1 + 2 (50 with low expression vs. 49 with high expression levels) notable differences were observed (16.0 months of average surveillance for the low expression cohort vs. 35.20 months for the high expression cohort, *p* = 0.0075) (Figure 1C). For patients in stages 3 + 4, no differences were detected (396 showing low expression vs. 605 showing high expression, *p* = 0.19) (Figure 1D). Accordingly, K-MPdb analysis was performed considering the grade of tumor. For grade 1 and 2, the group of patients with high expression of *ADORA2B* shown a major survival probability than those with lower expression level (*p* = 0.03 and *p* = 0.05 for grades 1 and 2, respectively); for grade 3, differences were not observed (*p* = 0.06); for grade 4, it was not possible to perform the analysis due to an insufficient number of patients (Supplementary Figure S2).

These data strongly suggested that low *ADORA2B* expression is a bad prognosis factor for serous ovarian carcinoma.

### 2.2. SKOV-3 Cell Line Expresses a Functional A2BR

**Figure 2:**
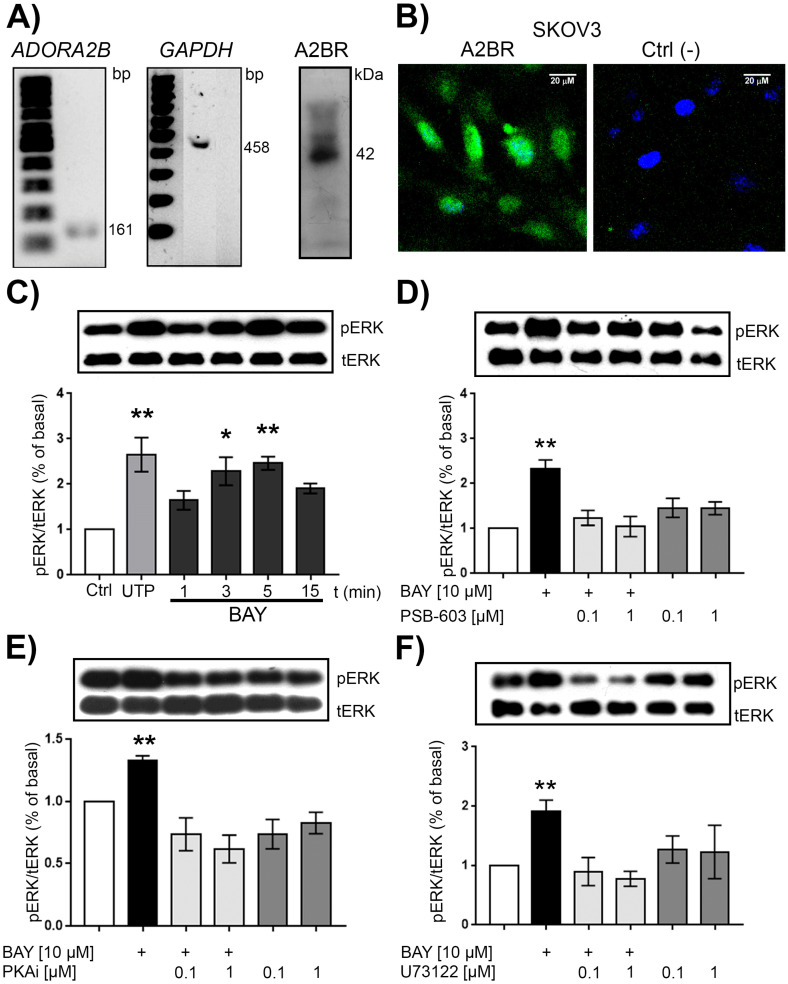
Ovarian carcinoma-derived SKOV-3 cells express functional A2BR receptors. (**A**) A fragment of the *ADORA2B* transcript was amplified by RT and PCR using specific oligonucleotides. An amplicon of 161 bp was obtained. *GAPDH* was used as a constitutive transcript (**left panels**). A2BR was detected by Western blot and immunoprecipitation of biotinylated membrane proteins from SKOV-3 cells; a main band of around 42 kDa was detected (**right panel**). (**B**) A2BR was labeled by immunofluorescence. The immunoreactivity was detected with a secondary antibody conjugated with AlexaFluor 488 (green signal). Nuclei were counterstained with DAPI (blue signal). (**C**) Cell cultures were stimulated with 10 μM of BAY-606583 for 1, 3, 5 or 15 min. Phosphorylated ERK (p-ERK) and total ERK (t-ERK) were detected by Western blot in the same membrane. As a positive control, UTP 100 μM was utilized. (**D**) ERK phosphorylation induced after 5 min of stimulation with BAY-606583 was prevented by the A2BR antagonist PSB-603; this effect was also inhibited by a PKA inhibitor (**E**) or by U73122, a PLC inhibitor (**F**). In the graphs, bars represent the mean ± S.E.M. of four independent experiments in duplicate. * *p* < 0.05, ** *p* < 0.01.

To analyze the cellular effects elicited by A2BR activity in ovarian carcinoma cells, we used the SKOV-3 cell line. Although, in previous work, our research group and others reported A2BR expression in this cell line, we confirmed this observation by RT-PCR and Western blotting of biotinylated membrane proteins. For RT-PCR, an expected band of 161 bp was obtained (Figure 2A, left panel). The amplicon was purified and sequenced; then, the sequence was analyzed in the BLAST platform (NIH-USA). The amplicon was identified with the entry NM_000676, corresponding to the *Homo sapiens* *ADORA2B* transcript (coding for A2BR). Glyceraldehyde-3-phosphate-dehydrogenase (*GAPDH*) transcript was used as a constitutive control (Figure 2A, central panel). 

Membrane proteins from SKOV-3 cells were biotinylated and isolated by immunoprecipitation with streptavidin-conjugated beads. The precipitates were analyzed by Western blot using an antibody directed against an extracellular epitope of A2BR as described in Methods. A main band of around 42 kDa was identified (Figure 2A, right panel) in accordance with the electrophoretic migration described by others [27]. Furthermore, A2BR immunostaining depicted that the receptor was distributed across the cell surface (Figure 2B). These results confirmed that A2BR is a membrane protein expressed in SKOV-3 cells. 

It is known that extracellular signal-regulated kinases (ERK) are A2BR effectors [28]. To test A2BR functionality, SKOV-3 cells were stimulated with 10 μM of BAY-606583 for different time intervals between 1 and 15 min, and ERK phosphorylation was evaluated by Western blot. The stimulus induced an increment in ERK phosphorylation that reached its maximum level after 5 min (2.40 ± 0.14% of basal) (Figure 2C). To support that this response was mediated by A2BR, cultures were preincubated (20 min) with 100 nM and 1 μM of the A2BR antagonist PSB-603 (PSB) before applying the BAY-606583 stimulus. PSB inhibited ERK phosphorylation induced by the agonist at both concentrations (1.30 ± 0.17% of basal at 100 nM and 0.90 ± 0.22% of basal at 1 μM) (Figure 2D).

It has been described that *ADORA2B* can couple both G*α*s and G*α*q proteins; to explore the pathway driving the A2BR-dependent ERK phosphorylation in SKOV-3 cells, we analyzed the effects of blocking the effectors of each pathway (PKA for G*α*s and PLC for G*α*q pathways). PKA was inhibited with the PKA inhibitor fragment 14–22 myristoylated (PKAi), whereas U73122 inhibited PLC. Both inhibitors were used at 100 nM and 1 μM. PKAi abolished the BAY-606583 (10 μM)-induced ERK phosphorylation (0.74 ± 0.13% and 0.62 ± 0.11% of basal at 100 nM or 1 μM of the inhibitor, respectively; Figure 2E). U73122 also inhibited ERK phosphorylation induced by BAY-606583 (0.90 ± 0.12% and 0.78 ± 0.13% of basal at 100 nM or 1 μM of the inhibitor, respectively, Figure 2F).

### 2.3. A2BR Stimulation Inhibited Cell Migration without Modifying Cell Proliferation

**Figure 3:**
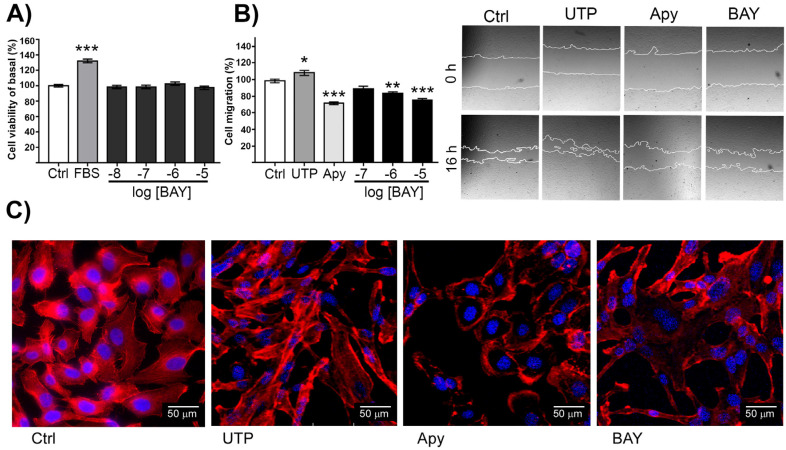
Stimulation of A2BR inhibits basal SKOV-3 cell migration but has no effect on cell viability. (**A**) Cultures of SKOV-3 cells at 50% confluence were stimulated for 24 h with BAY-606583 from 10 nM to 10 μM; then, cell viability was estimated by MTS assay. (**B**) Confluent cultures of SKOV-3 cells were stimulated for 16 h with BAY-606583 from 100 nM to 10 μM, and cell migration was estimated by scratch assay. UTP (100 μM) and Apy were used as positive and negative controls, respectively. (**C**) SKOV-3 cells were treated for 16 h with 100 μM UTP, 10 U/mL Apy or 10 μM BAY-606583; then, the actin cytoskeleton was stained with rhodamine-coupled phalloidin, and nuclei were counterstained with DAPI. In the graphs, bars represent the mean ± S.E.M. of four independent experiments in sextuplicate for A and in triplicate for B. * *p* < 0.05, ** *p* < 0.01, *** *p* < 0.001.

A central aim of this project was to evaluate the effects of A2BR activity on the phenotype of ovarian carcinoma-derived cells; thus, cell viability and cell migration were analyzed after pharmacological stimulation of this receptor. SKOV-3 cells were stimulated for 24 h with increased concentrations (from 10 nM to 10 μM) of BAY-606583 in serum-free media. We used 10% fetal bovine serum (FBS) as a positive control for the experiment; after the stimulus, cell viability was evaluated applying the MTS method. FBS induced an increment in cell viability (132.70 ± 2.40% of control, *p* < 0.05), whereas BAY-606583 did not induce changes at any of the evaluated concentrations (Figure 3A).

To test if A2BR regulates cell migration, SKOV-3 cells were stimulated with 100 nM, 1 μM and 10 μM of BAY-606583 and migration was estimated by scratch assay after 16 h. Based on a previous report, we used UTP as a positive control for cell migration and Apy to inhibit cell migration [29]. UTP promoted SKOV-3 cell migration (108.1 ± 3.0) and Apy reduced this parameter (71.5 ± 1.6% of control, *p* < 0.05; Figure 3B). Interestingly, BAY-606583 100 nM, 1 μM and 10 μM also reduced cell migration (88.7 ± 3.5%, 83.3 ± 2.0% and 75.7 ± 1.6%, respectively, *p* < 0.05) (Figure 3B). 

Furthermore, to observe stress fibers (SF), which indicate a mesenchymal-like phenotype, the actin cytoskeleton was labeled with phalloidin coupled to rhodamine. As controls, UTP increased the SF, whereas Apy treatment notably reduced them. In addition, the presence of SF in SKOV-3 cells treated with BAY was less evident than in control cells (Figure 3C, red signal). 

To investigate if migration in other ovarian carcinoma-derived cell lines was also modulated by A2BR, CAOV-3 cells, a cellular line derived from a primary tumor of ovarian carcinoma, were incubated with the A2B receptor agonist BAY-606583 (at 0.1, 1 and 10 μM) by 16 h, and cell migration was evaluated by scratch assay. A2BR activation inhibited cell migration at all the concentrations tested; moreover, expression of *ADORA2B* transcript was confirmed by RT-PCR (Supplementary Figure S3).

**Figure 4:**
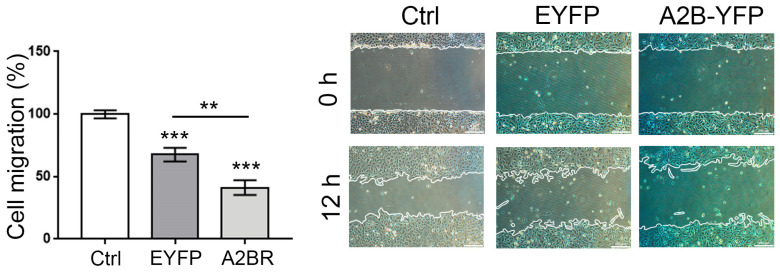
Overexpression of A2BR inhibits basal migration of SKOV-3 cells. SKOV-3 cells were transfected with a plasmid coding for A2BR fused with yellow fluorescent protein (YFP) at the carboxy-end; another plasmid coding only for YFP was used as a control. After transfection, cells were cultured to reach confluence (48 h) and a scratch assay was performed. Pictures show the wound at the time it was made and 16 h after. In the graphs, bars represent the mean ± S.E.M. of three independent experiments. ** *p* < 0.05, *** *p* < 0.01. Scale bar = 250 µm

**Figure 5:**
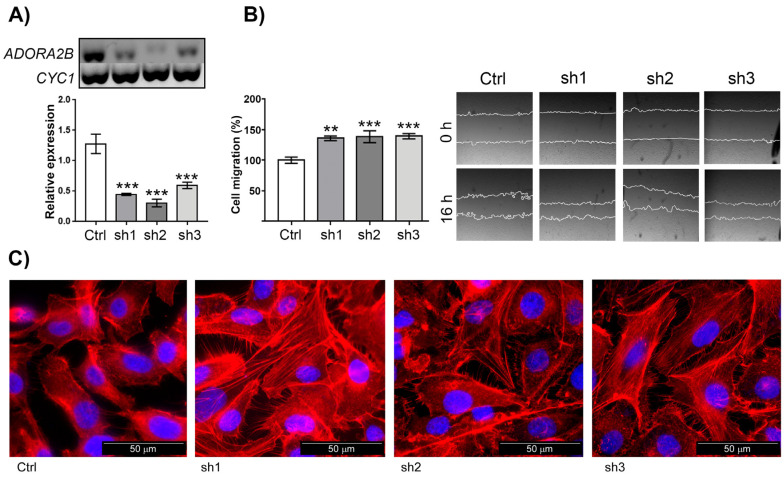
Knockdown of the *ADORA2B* transcript induced an increment in basal cell migration and in stress fiber formation. (**A**) Cultures of SKOV-3 cells were transduced with lentiviral particles carrying three different shRNAs directed against the *ADORA2B* transcript. The effect of knockdown was monitored by RT-PCR; *CYC1*, coding for a subunit of cytochrome bc1 complex, was used as a constitutive transcript. Relative expression was analyzed by agarose gel electrophoresis densitometry. (**B**) Scratch assay was performed to estimate the cellular migration of control or *ADORA2B*-shRNA expressing SKOV-3 cells (sh1 to sh3), 16 h after the wound was made. In the graphs, bars represent the mean ± S.E.M. of three independent experiments. ** *p* < 0.05, *** *p* < 0.01. (**C**). The actin cytoskeleton of control or *ADORA2B*-shRNA expressing SKOV-3 cells was stained with rhodamine-coupled phalloidin and nuclei were counterstained with DAPI.

To further support the observation that A2BR attenuates the mesenchymal characteristics of SKOV-3 cells and their concomitant cell migration capacity, its expression was modified in two ways: (1) by overexpression and (2) by knockdown with shRNAs. A2BR overexpression induced an inhibition of cell migration (41.24 ± 5.96%, Figure 4), whereas A2BR knockdown with three different shRNAs resulted in enhanced cell migration (135.80 ± 3.72, 138.40 ± 9.62 and 139.20 ± 4.36 % of control for shRNAs 1, 2 and 3, respectively, *p* < 0.05%) (Figure 5A,B). Visualization of the actin cytoskeleton by phalloidin-rhodamine staining revealed that the knockdown of the *ADORA2B* transcript induced an increment in the presence of SF (Figure 5C). Taken together, the evidence suggests that A2BR was not involved in cell proliferation, but it negatively regulated cell migration.

### 2.4. ADORA2B Enhances the Expression of the Epithelial Marker E-Cadherin in SKOV-3 Cells

**Figure 6:**
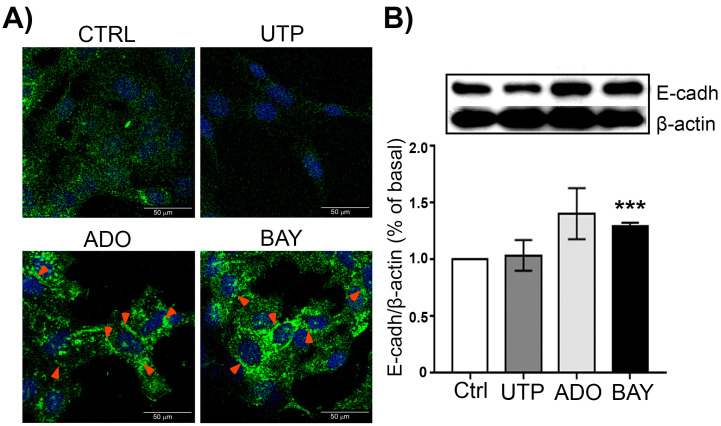
Stimulation of A2BR with BAY-606583 incremented the expression and induced the relocation of E-cadherin. SKOV-3 cell cultures were stimulated for 16 h with 10 μM BAY-606583, 10 μM ADO or 100 μM UTP. E-cadherin was detected by immunofluorescence (**A**) using a specific primary antibody and a secondary one coupled to AlexaFluor 488 (green signal). Nuclei were counterstained with DAPI (blue signal) and Western blot (**B**), where the abundance of E-cadherin was expressed in relation with against β-actin as housekeeping protein. In the graph, bars represent the mean ± S.E.M. of three independent experiments, *** *p* < 0.01 vs. Ctrl.

To analyze whether the reduction in cell migration induced by A2BR activation is associated with phenotypical changes in SKOV-3 cells, we performed immunofluorescence labeling and Western blot analysis of the epithelial marker E-cadherin in SKOV-3 cells treated with BAY-606583. In control conditions, E-cadherin was absent from the cell junctions and showed a discreet cytoplasmic pattern (Figure 6A). In contrast, the cultures stimulated with BAY-606583 and ADO showed a significant increase in the expression of E-cadherin in the cell junctions. As a negative control of E-Cadherin up-regulation, we incubated the cells with UTP, a nucleotide that induced the mesenchymal phenotype [29]; as expected, UTP did not elicit changes in E-cadherin level or location (Figure 6A). These observations were reinforced by Western blot assays, as BAY-606583 induced an augmentation in the level of E-cadherin (1.29 ± 0.03%) (Figure 6B).

### 2.5. Gene Expression Mediated by A2BR Activation in SKOV-3 Cells

To analyze the transcriptional regulation promoted by A2BR activation, and its relationship with cell migration, we stimulated SKOV-3 cells with BAY-606583 10 μM for 24 h; then, the total RNA was isolated, labeled and hybridized with a 35k library from the whole human genome.

**Figure 7:**
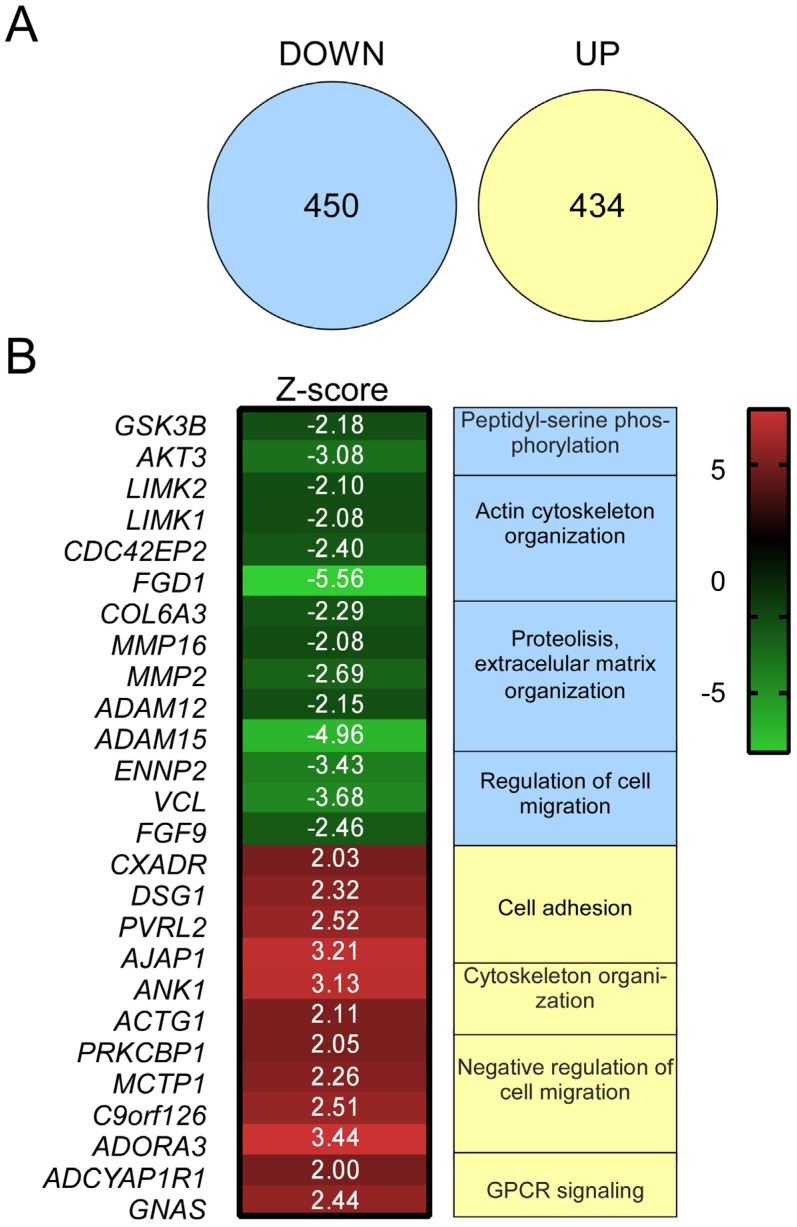
Regulation of gene expression patterns by A2BR activation with BAY-606583 in SKOV-3 cells was analyzed by cDNA microarrays. SKOV-3 cell cultures were stimulated with 10 μM BAY-606583 for 16 h and total RNA was isolated; a pool of five independent cultures was hybridized against a library of 30 K genes of the complete human genome. (**A**) BAY-606583 induced broad changes in gene expression; the Venn diagram represents the set of transcripts up- or down-regulated by the agonist. (**B**) A heat-map representation of the Z-score of outstanding transcripts up- or down-regulated by BAY-606583. The cellular process determined by GO analysis is shown.

We observed that A2BR activity induced substantial changes in the gene expression profile of SKOV-3 cells: from the 884 modified transcripts, 450 were down-regulated and 434 up-regulated (Figure 7A). 

Analysis of the down-regulated genes revealed transcripts grouped in the following categories: *Regulation of Cell Migration*, *Proteolysis and Extracellular Matrix Organization* and *Peptidyl-Serine Phosphorylation* (Figure 7B). Among the most interesting down-regulated transcripts, it was observed *FGF9*, *AKT3*, *GSK3b* and the metalloproteinases *ADAM12*, *MMP2* and *MMP16*, all related with cell invasiveness (Table 1). On the other hand, GO analysis showed up-regulation of transcripts in the categories *Negative Regulation of Cell Migration*, *Cytoskeleton Organization* and *Cell Adhesion* (Figure 7B). Some relevant transcripts up-regulated were *C9orf126* (*SCAI*), *PRKCBP1* (*ZMYND8*) and *PVRL2* (Nectin 2), related with negative regulation of cell migration (Table 1). More detail is developed in the Discussion section.

## 3. Discussion

Ovarian carcinoma is one of the most lethal gynecologic malignancies. Most patients are diagnosed in advanced stages, explaining in part its high mortality rate. Therefore, it is important to study the mechanisms that enable ovarian cancer cells to become metastatic. ATP and ADO are major components of the TME [2,30]. Both molecules have proved to modulate cancer cell migration and EMT by acting through purinergic receptors [20]. In previous works, we have shown that ADO and NECA, an agonist of A2 receptor with preference to A2BR, favors an epithelial phenotype and decreases SKOV-3 cell migration [24]. Moreover, A2BR is the most expressed ADO receptor [24], suggesting that it plays a key role in the modulation of ovarian cancer cell migration.

By using public K-MPdb (ovarian cancer) we detected a positive correlation between higher expression of A2BR and the survival rate of patients (Figure 1A and Supplementary Figure S2). This correlation was observed in serous ovarian carcinoma (Figure 1B); in stages 1 + 2, when the tumor is yet confined to the primary tumor, but not in stages 3 + 4, when cancer showed dissemination (Figure 1C,D); in agreement, the same correlation was observed in patients with serous ovarian carcinoma grade 1 and 2, when tumor cells are not yet undifferentiated, but not in grade 3 (Supplementary Figure S2). Thus, high expression of *ADORA2B* at early stages of ovarian cancer could be associated with good prognosis. However, it is pertinent to mention that we were only able to perform the analysis for serous subtype of OvCa because this was the only pathological subtype with the necessary number of patients in the database.

An explorative analysis of K-MPdb in other cancers (supplementary Figure S1), showed that for lung carcinoma a high expression level of *ADORA2B* transcript is associated with bad prognosis, in opposition with ovarian carcinoma; while for breast and gastric carcinoma, no differences between low or high level of the transcript were observed, suggesting that the contribution of A2B receptor to patient survival is tissue-specific and cancer subtype-specific. These observations justify the characterization of A2B receptor in particular cell lines. Hence, to understand if the autocrine-paracrine activation of A2BR contributes to ovarian cancer-derived cell physiology, we used SKOV-3 cells.

To analyze the possible role of A2B receptor in cell migration, first, A2BR expression and functionality was confirmed by the following evidence: (1) The transcript of the *ADORA2B* gene was detected by RT-PCR (Figure 2A), amplicon identity was confirmed by sequencing; (2) A2BR expression in the plasma membrane of SKOV-3 cells was shown by Western blot analysis of proteins labeled by biotinylation with a non-permeant reactive and isolated by immunoprecipitation with avidin-coupled beads (Figure 2A); (3) concentration-dependent ERK phosphorylation in response to BAY-606583, a specific A2BR agonist, was blocked by the selective antagonist PSB-603 (Figure 2C,D); (4) BAY-606583-induced ERK phosphorylation was inhibited by PKAi fragment 14–22 myristoylated and by U-73122, a PLC inhibitor, strongly suggesting that A2BR is coupled to both Gαs and Gαq subunits in SKOV-3 cells (Figure 2E,F). This latter observation is interesting because A2BR signaling mechanisms are complex and must be delineated in each experimental model. For instance, in human epithelial lung cancer A2BR can couple to both subunits and, depending on the activated cell signaling pathway, can elicit different physiological processes [23]. Moreover, Gαs coupling has been demonstrated in breast cancer cells (MDA-MB-231) [18]. 

Then, we aimed our studies to evaluate the influence A2B receptor has on cell migration given that this process is determinant for cancer outcome. From our studies we conclude that A2BR activation in SKOV-3 cells inhibited cell migration. The evidence was as follows: (1) activation of A2BR with BAY-606583 (100 nM to 10 μM) induced a reduction in cell migration evaluated by the scratch assay (Figure 3B); (2) this result correlated with less-marked SF when cells were treated with A2BR agonist or Apy (Figure 3C), indicating an attenuated mesenchymal phenotype; (3) A2BR overexpression reduced cell migration (this result was similar to the one observed with pharmacological stimulation of the receptor (Figure 4)); (4) the reduction in A2BR expression by three different shRNAs caused an increase in cellular migration (Figure 5B) (this effect was in parallel with an increment in SF (Figure 5C)). Altogether, our data suggests that A2BR activation or constitutive activity inhibits SKOV-3 cell migration. To discard the idea that migration experiments were interfered with by cell proliferation, we evaluated if A2BR regulated cell viability, and found that treatment with BAY-606583 for 24 h had no effect on cell viability (Figure 3A), indicating that migration experiments are not influenced by proliferation. Furthermore, analysis of E-cadherin expression and localization pattern in response to BAY-606583 (Figure 6), indicated that A2BR stimulation favored mesenchymal phenotype. Similar observations were described when cervical cancer cells were treated with ADO [22]. 

In agreement with these observations, BAY-606583 also inhibited migration of CAOV-3 cells, another line derived from ovarian carcinoma, where A2BR is also expressed (Supplementary Figure S2). These data are suggestive that the actions of A2BR on ovarian carcinoma cell migration could be a more general mechanism.

To consider the implications of our observations in ovarian cancer, it is necessary to take into account that the role of A2BR on cell migration depends on the cellular model studied. Thus, it was shown that A2BR activation in breast [18] urothelial; [17] and head and neck squamous cell carcinoma [31] increased cell migration. On the other hand, in human cervical cancer cells, extracellular ADO inhibited migration and reduced invasion [22] and, in noncancerous human retinal epithelial pigment cells, A2BR activation also inhibited cell migration [32]. Moreover, in epithelial lung cancer cells [23] and in mouse mammary fibroblast [33], A2B activity negatively modulated cell migration and the induction of markers of the epithelium to mesenchymal transition, counteracting the actions of TGF-β [23,33]. 

Thus, the inhibition of cell migration observed in the present study could contribute to explain the correlation between a high expression level of *ADORA2B* and a higher probability of patient survival at initial stages of the disease (Figure 1). However, there is at least another possibility; because Skov-3 cells are metastatic, the inhibition of migration and favoring of epithelial phenotype could contribute to cancer colonization of secondary organs, where cells display mesenchymal to epithelial transition (MET) [34]. Since cancer cells secretes exosomes expressing CD39 and CD73, enzymes necessary to convert ATP in ADO [35,36], is plausible to hypothesize that ADO acting through A2BR could be contributors to secondary tumor formation. 

Finally, to understand if A2BR activity promoted changes in gene expression patterns and if these changes regulated cell migration, transcriptional activity of stimulated SKOV-3 cells was analyzed by cDNA microarrays. Indeed, pharmacological A2BR activation induced important changes in gene expression patterns. The main categories identified by GO analysis in down-regulated transcripts were Proteolysis and Extracellular Matrix Organization, Actin Cytoskeleton Organization and Regulation of Cell Migration, while in the up-regulated transcripts, the principal categories were Cell Adhesion, Negative Regulation of Cell Migration and Cytoskeleton Organization (Figure 7, Table 1). Fibroblast growth factor 9 (*FGF9*) stands out from the down-regulated transcripts. It has been reported that FGF9 is able to induce ovarian cancer cell invasion by activating the VEGF-A/VEGFR2 pathway [37]. Other transcripts included *AKT3* and *GSK3β*, coding for the variable 3 of protein kinase b and for Glycogen synthase kinase 3 beta, respectively; both factors induce cell proliferation and migration in ovarian carcinoma cells [38,39]. Of notable interest was the down-regulation of transcripts coding for metalloproteinases including *ADAM12*, a factor associated with an aggressive phenotype in high-grade serous ovarian carcinoma [40]. In addition, *MMP2* and *MMP16* transcripts, which are recognized proteins mediating enhanced migration and metastasis of ovarian carcinoma cells, were also reduced [41,42].

Some outstanding up-regulated transcripts were *C9orf126*(*SCAI*), *PRKCBP1* (*ZMYND8*), and *PVRL2* (Nectin 2). *C9orf126*(*SCAI*) codes for suppressor of cancer cell invasion protein (SCAI protein), a component of the RhoA signal transduction pathway. It has been shown in non-small lung cancer cells that miR-371b-5p induces cell proliferation, migration and invasion by negatively regulating SCAI expression [43]. *PRKCBP1* (*ZMYND8*) codes for zinc finger MYND-type containing 8, a transcription factor and histone-interacting protein that regulates cellular growth. In cancer cells, ZMYND8 modulates histone methylation and acetylation, regulating the expression of oncogenes and tumor suppressors [44]. In breast and nasopharyngeal cancers, ZMYND8 is down-regulated and its low expression correlates with increased invasiveness and poor prognosis [45,46]. Another interesting up-regulated transcript was *PVRL2* (Nectin 2), an adhesion protein participating in the initial step of cell-to-cell adherens junctions [47,48] that can also interact with scaffold proteins to regulate cell movement and differentiation [49]. It has been proposed that Nectin-2 is a potential target for breast and ovarian cancers [50].

Taken together, our results suggest that elevated levels of A2BR could be associated with a good prognosis in early ovarian cancer stages (1 + 2). Our in vitro results indicate that A2BR pharmacological stimulation and overexpression decreased ovarian cancer cell migration, which is associated with the acquisition of an epithelial phenotype. 

## 4. Materials and Methods

### 4.1. Cell Culture

SKOV-3 (HTB-77) cells were acquired from the American Type Culture Collection (ATCC, Manassas, VI, USA). Cells were maintained in RPMI medium supplemented with 10% fetal bovine serum (FBS) and 1X antibiotic–antimycotic solution (for each mL: penicillin 100 U, streptomycin 100 μg and fungizone 0.25 μg) (Thermo Scientific, Waltham, MA, USA) at 37 °C in a humidified 5% CO_2_ atmosphere. All reagents were obtained from Gibco-Thermo Fisher Scientific, USA. 

### 4.2. Reverse Transcription (RT) and Polymerase Chain Reaction (PCR)

Total RNA was isolated following the guanidine isothiocyanate method [51]. RNA integrity was evaluated by electrophoresis and its concentration was determined by spectrophotometric analysis (NanoDrop 1000, Wilmington, DE, USA). cDNA was synthesized with 1 μg of total RNA treated with DNAse activity-free RNAse and used for reverse transcription reaction. The mixture contained oligodT and Moloney Murine Leukemia Virus (M-MLV) reverse transcriptase (Promega, Madison, WI, USA). Amplification of the transcripts coding for A2BR (*ADORA2B*), glyceraldehyde 3-phophate dehydrogenase (*GAPDH*) and cytochrome C1 (*CYC1*) was performed by end-point PCR. The oligonucleotide sequences were as follows: A2BR-forward 5′-TCC ATC TTC AGC CTT CTG GC-3′, A2BR-reverse 5′-AAA GGC AAG GAC CCA GAG GA-3′; GAPDH-forward 5′-CAA GGT CAT CCA TGA CAA CTT TG-3′, GAPDH-reverse 5′-GTC CAC CAC CCT GTT GCT GTA G-3′ and CYC-1-forward 5′-CTC CTG CCA CAG CAT GGA C-3′, CYC1-reverse 5′-CAT GCC TAG CTC GCA CGA T-3′. Reactions were performed in a final volume of 20 μL. All primers were synthesized by Sigma-Aldrich, USA. End-point PCR reactions were performed in a BioRad thermocycler. Amplicon identity was corroborated by sequences and BLAST (NIH) analysis. 

### 4.3. Biotinylation of Plasma Membrane Proteins

For biotinylation of proteins located in the plasma membrane of SKOV-3 cells, cultures at 80–90% confluence were incubated with 300 μM of Ez-link Sulfo NHS-LC-LC-Biotin reagent (Thermo Scientific, Waltham, MA, USA) diluted in phosphate buffer (PBS, in mM:136 NaCl, 2.7 KCl, 10 Na_2_HPO4, 1.8 KH_2_PO4, pH 7.4) for 20 min. Then, biotinylation solution was withdrawn and cells were washed twice with PBS to be solubilized in TNTE buffer (containing in mM: 50 Tris–HCl pH 7.4, 150 NaCl, 1 EDTA, and 0.1% Triton X-100) for 20 min on ice. Cellular homogenate was collected and centrifuged at 10,000 rpm for 10 min at 4 °C. The supernatant was recovered, and the pellet discarded. Protein concentration of the cellular extract was estimated by the Lowry method and 1 mg of protein was incubated with 50 μL of sepharose–streptavidin-conjugated beads (Cell Signaling Technology, Danvers, MA, USA) for 90 min at room temperature. After, the beads were washed 3 times with PBS and resuspended in 100 μL of Laemmli buffer, boiled for 5 min and analyzed by Western blot as described below. For A2BR detection an antibody against an extracellular epitope (KDSATNNSTEPWDGTTNESC) of the receptor was employed (Allomone Labs, Jerusalem, Israel; #AAR-003) at a 1:1000 dilution.

### 4.4. Western Blot

Equal numbers of cells were seeded in 12-well plates. When the cells reached 80% confluency, they were serum-starved overnight. Then, pharmacological treatment was performed at the concentration and time indicated in each experiment. Cell lysates were obtained using Laemmli solution. Electrophoresis was made in 10% or 8% SDS-polyacrylamide gels and cells were transferred to PVDF membranes. For detection, membranes were incubated overnight at 4 °C with the following primary antibodies in 1:1000 dilution: anti-phospho p44/42 MAPK, anti-total p42/p44 MAPK, anti-E-cadherin (Cell signaling Technologies, Danvers, MA, EUA) or anti-A2BR (Alomone Labs, Jerusalem, Israel). Antibodies were raised in rabbit and anti-E-cadherin in mouse. After primary antibody incubation, membranes were incubated for 1 h with conjugated horseradish peroxidase goat anti-rabbit or donkey anti-mouse antibodies at a 1:5000 dilution. Signal was revealed by chemiluminescence and autoradiography. Densitometry analysis was performed using ImageJ software. 

### 4.5. Cell Viability 

MTS (3-(4-5-dimethylthiazol-2-yl)-5-(3-carboxymethoxyphenyl)-2-(4-sulfophenyl)-2 -H- tetrazolium) salt reduction assay (Cell Titter, Promega, USA) was used to evaluate cell viability. SKOV-3 cells (3.5 × 10^3^) were cultured in 48-well plates in RPMI medium supplemented with 10% FBS to reach 50% confluence, then they were starved and incubated for 12 h. A2BR was stimulated by the addition of BAY-606583 from 10 nM to 10 μM and incubated for an additional 24 h. MTS reduction was determined according to the manufacturer’s protocol. Briefly, MTS reactive was added to the cell culture at a final concentration of 16.6%, incubated for 2 h under standard culture conditions, recollected in a clean 96-well dish and read in a spectrophotometer at 490 nM. Data were normalized against control (non-stimulated condition). The assay was repeated four times in septuplicates.

### 4.6. Lentiviral Infection

Lentiviral particles were generated by transfecting HEK293-T cells with 10 μg of pLKO.1 plasmid carrying one of the anti-*ADORA2B* shRNA: sh1 5′-CCG GGC AGA TGT CAA GAG TGG GAA TCT CGA GAT TCC CAC TCT TGA CAT CTG CTT TTT G-3′ (SIGMA #TRCN0000065335); sh2 5′-CCG GGC AAT GAA TAT GGC CAT TCT TCT CGA GAA GAA TGG CCA TAT TCA TTG CTT TTT G-3′ (SIGMA # TRCN0000065337) or sh3 5′-CCG GGC TGG TGA TCT ACA TTA AGA TCT CGA GAT CTT AAT GTA GAT CAC CAG CTT TTT G-3′, (SIGMA #TRCN0000065334) (SHCLNG_NM000676; Sigma-Aldrich) and the packaging vectors pRSV-Rev, pMD2.G and pMDLg/pRRE, by the calcium phosphate precipitation method. Supernatant HEK293-T medium was collected and used to transduce SKOV-3 cells. Transduced cells were selected by puromycin (1.5 μg/mL) resistance for 5–7 days. Monitoring of *ADORA2B* knockdown was made by RT-PCR as described above.

### 4.7. Immunofluorescence 

Immunostaining was performed as previously described [52]. Briefly, SKOV-3 cells were cultured on coverslips and pharmacological treatments were applied if necessary. For immunodetection, cells were washed with PBS, fixed for 20 min in a PBS solution containing 4% paraformaldehyde (PFA), washed with PBS for 5 min, permeabilized with 0.01% Triton X-100 in PBS and blocked with 5% fat-free milk in PBS for 1 h. Then, cells were incubated overnight with primary antibodies against E-cadherin (1:100; mouse; Cell Signaling) or A2BR (1:100; rabbit; Allomone). The next day, samples were washed twice with PBS and incubated for 1 h with secondary antibodies, anti-rabbit IgG coupled to Alexa 488 or anti-mouse IgG coupled to Alexa 488 (Thermo Scientific). Finally, the samples were mounted with VectaShield containing 4′, 6′-diamidino-2-phenylindole (DAPI) (Vector, Burlingame, CA, USA). The samples were analyzed by confocal microscopy (LSM-780 Carl Zeiss). 

### 4.8. Actin Cytoskeleton Labeling

Wild-type or *ADORA2B* knockdown SKOV-3 cells were cultured on coverslips. After the indicated pharmacological treatment, they were fixed and permeabilized as described for immunostaining. Then, cells were incubated for 10 min in a PBS-DAPI solution (dilution 1:1000), washed, and mounted in VectaShield containing phalloidin coupled to rhodamine (Vector, CA, USA). The samples were analyzed by fluorescence microscopy (Apotome, Carl Zeiss, Jena, Germany).

### 4.9. Wound Closure Assay

Cells were cultured on 12-well plates. When the cultures reached 90% confluence, they were starved for 16 h. Then, a wound was made along the culture well using a 200 μL pipette tip, and cells were incubated with the indicated pharmacological treatment for another 16 h. Pictures were taken just after pharmacological stimulation (t = 0) and after 16 h. Analysis of the wound closure area was performed using microphotographs and Image J software. All wound closure experiments were repeated at least three times in triplicate.

### 4.10. Cancer Database Analysis

The Kaplan–Meier plotter database (https://kmplot.com/analysis/; accessed on 1 January 2022) was used to generate survival curves from ovarian cancer patients [26]. Log rank P, hazard ratio and median survival or upper quartile survival were calculated and displayed on the webtool. 

### 4.11. cDNA Microarray Analysis

The cDNA microarray experiment was performed in the Microarray Unit at the Institute of Cellular Physiology (Universidad Nacional Autónoma de México, CDMX, Mexico). SKOV-3 cells were cultured in 100 mm Petri dishes to achieve a confluence of 70–80%. Then, cells were stimulated with BAY-606583 for 24 h or kept under control conditions. Later, total RNA was isolated by the Trizol method (Thermo Fisher Scientific, Waltham, MA, USA) and used for cDNA synthesis with a commercial kit using 10 mg of total mRNA (First-Strand cDNA labeling kit, Thermo Fisher Scientific, Waltham, MA, USA); dUTP-Alexa555 or dUTP-Alexa647 probes were incorporated at this stage. Fluorescence emission was analyzed at 555 nm for Alexa555 and 650 nm for Alexa647.

Labeled cDNA was utilized to hybridize a 35 K library of the whole human genome containing 70-mer oligos (from OPERON) manufactured by the Microarray Unit of the Institute of Cellular Physiology at UNAM. The acquisition and quantification of the array images was performed with GenePix 4100A software (OMICtools, RRID:SCR_002250; Molecular Devices, San José, CA, USA). Mean density values for the fluorescent probes and mean background were calculated. Analysis of the microarray data was performed with genArise (RRID:SCR_001346; http://www.ifc.unam.mx/genarise/; accessed on 1 August 2021) developed by the Computing Unit of the Institute of Cellular Physiology (UNAM, Mexico). This freeware calculates the intensity-dependent Z-score from the images to identify different gene expression patterns. Elements with a Z-score > 1.5 standard deviations were defined as differentially expressed transcript genes. Data were deposited in ArrayExpress-EMBL-EBI (accession number: E-MTAB-11130). Bioinformatic analysis was performed with GeneCodis and KEGG tools and focused on identifying genes regulated by BAY-606583 with a gene ontology focus. 

### 4.12. Statistics Analysis 

The results are expressed as the mean standard error of the mean (S.E.M.). The statistical differences between the groups were evaluated with a Student’s t-test and marked with * for *p* ≤ 0.05, ** for *p* ≤ 0.01 * and *** for *p* ≤ 0.001.

## Figures and Tables

**Table 1:** Transcripts regulated by A2BR stimulation with 10 mM of BAY for 24 h.

Down-Regulated Transcripts			
Gene symbol	Gene name	Description	Z score
*AKT3*	AKT serine/threonine kinase 3	Peptidyl-serine phosphorylation	−3.08
*GSK3B*	Glycogen synthase kinase 3 beta		−2.18
*FGF9*	Fibroblast Growth Factor 9	Regulation of cell migration	−2.46
*VCL*	Vinculin		−3.68
*ENNP2*	Ectonucleotide pyrophosphatase/phosphodiesterase 2		−3.43
*ADAM15*	ADAM Metallopeptidase Domain 15	Proteolysis, extracellular matrix reorganization	−4.96
*ADAM12*	ADAM Metallopeptidase Domain 12		−2.15
*MMP2*	Matrix Metallopeptidase 2		−2.69
*MMP16*	Matrix Metallopeptidase 16		−2.08
*COL6A3*	Collagen, Type VI, Alpha 3		−2.29
*FGD1*	FYVE, RhoGEF and PH domain containing 1	Actin cytoskeleton organization	−5.56
*CDC42EP2*	CDC42 effector protein 2		−2.40
*LIMK1*	LIM domain kinase 1		−2.08
*LIMK2*	LIM domain kinase 2		−2.10
*GNAI2*	Guanine Nucleotide-Binding Protein G(I) Subunit Alpha-2	G protein-coupled receptor signaling pathway	−2.50
*PDE3B*	Cyclic GMP-Inhibited Phosphodiesterase B		−4.49
*PDE9A*	Phosphodiesterase 9A		−2.99
**Up-Regulated Transcripts**			
Gene symbol	Gene name	Description	Z score
*GNAS*	Adenylate Cyclase-Stimulating G Alpha Protein	G protein-coupled receptor signaling pathway	2.44
*ADCYAP1R1*	ADCYAP receptor type I		2.00
*ARHGEF7*	Rho Guanine Nucleotide Exchange Factor 7		2.20
*PFN1*	Profilin 1	Actin cytoskeleton organization	6.10
*CDC42BPA*	CDC42 Binding Protein Kinase Alpha		2.31
*ADORA3*	Adenosine A3 Receptor	Negative regulation of cell migration	3.44
*C9orf126*	Suppressor Of Cancer Cell Invasion		2.51
*MCTP1*	Multiple C2 And Transmembrane Domain Containing 1		2.26
*PRKCBP1*	Protein Kinase C Beta		2.05
*ACTG1*	Actin Gamma 1	Cytoskeleton organization	2.11
*ANK1*	Ankyrin 1		3.13
*AJAP1*	Adherents Junctions Associated Protein 1	Cell adhesion	3.21
*PVRL2*	Nectin Cell Adhesion Molecule 2		2.52
*DSG1*	Desmoglein 1		2.32
*CXADR*	CXADR Ig-Like Cell Adhesion Molecule		2.03

## Data Availability

Microarray data were deposited in ArrayExpress-EMBL-EBI (accession number: E-MTAB-11130). Other data supporting the results of the current study are available from the corresponding author on reasonable request.

## Supplementary Materials

The following supporting information can be downloaded at: https://www.mdpi.com/article/10.3390/ijms23094585/s1.
