# Supplementary material for: Adenosine Receptor A2B Negatively Regulates Cell Migration in Ovarian Carcinoma Cells
Source: Int J Mol Sci. 2022 Apr 21;23(9):4585. doi: 10.3390/ijms23094585 (PMC9100769; doi:10.3390/ijms23094585)
Supplement: Supplementary file 1 [file ijms-23-04585-s001.zip › ijms-1654211-supplementary.pdf]

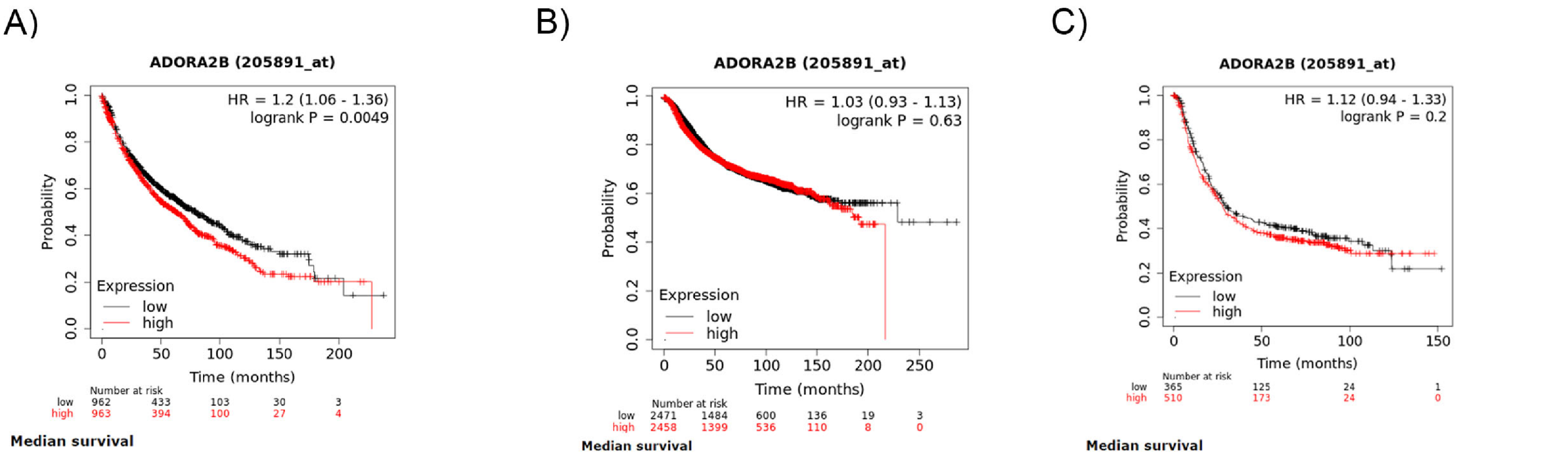

Median survival

Low expression cohort (months)	High expression cohort (months)
79.54	63

Median survival

Low expression cohort (months)	High expression cohort (months)
228.85	191.21

Median survival

Low expression cohort (months)	High expression cohort (months)
30.4	28.1

Supplementary Figure S1. ADORA2B expression level has tissue-specific relation with patient survivance. Kaplan-Meier plots for: (A) Lung, (B) Breast or (C) Gastric carcinomas.

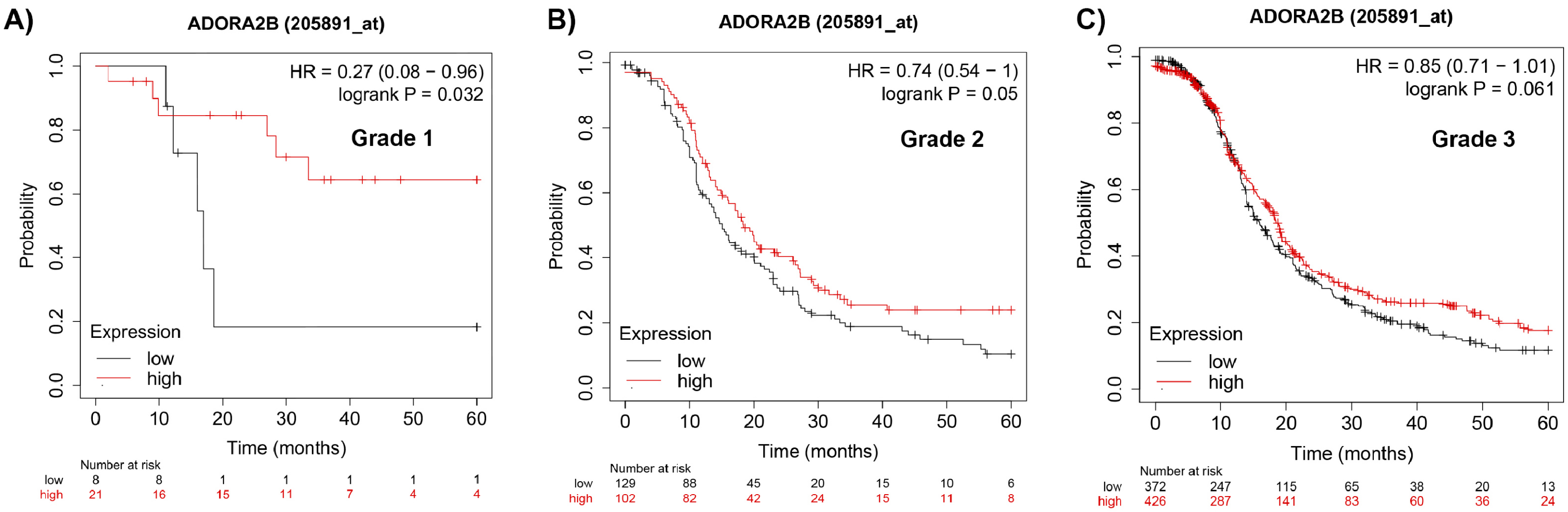

Upper quartile survival

Low expression cohort (months)	High expression cohort (months)
12.23	28.37

Median survival

Low expression cohort (months)	High expression cohort (months)
15	18.37

Median survival

Low expression cohort (months)	High expression cohort (months)
16.03	18.79

Supplementary Figure S2. Relation between ADORA2B transcript expression level and survival probability in serous ovarian cancer patients with tumors of different grades of the disease. Kaplan Meier plots were made using ADORA2B transcript as previously mentioned for (A) Ovarian serous carcinoma patients in grade 1 (21 high expression and 8 low expression), (B) Serous ovarian carcinoma in grade 2 (102 high expression and 129 low expression), and (C) Ovarian serous carcinoma patients in grade 3 (426 high expression and 372 low expression).

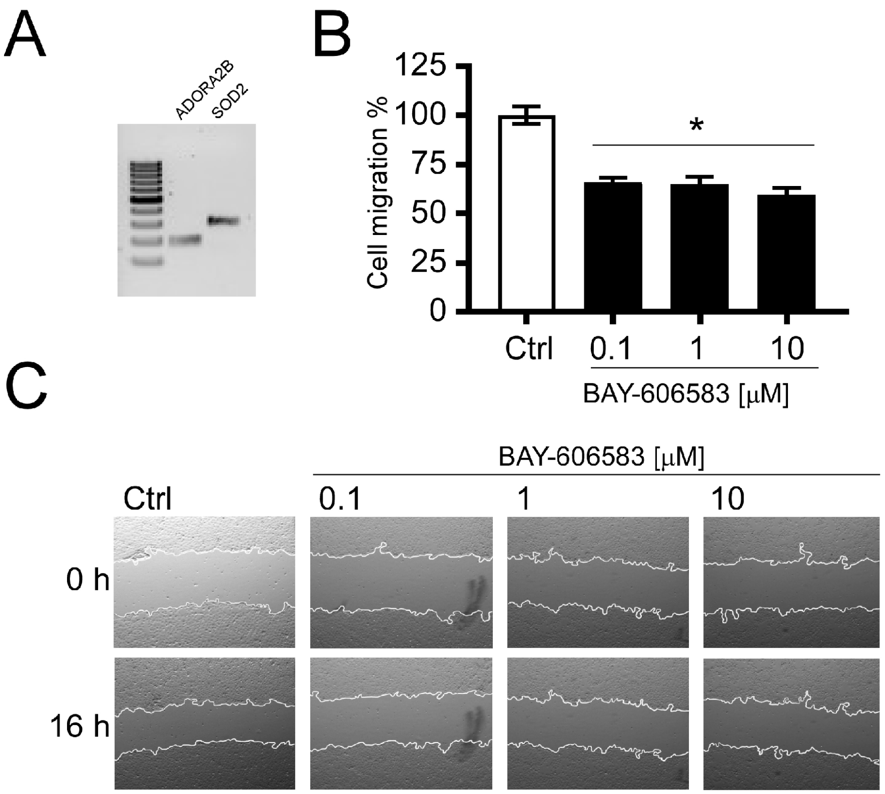

Supplementary Figure S3. Ovarian carcinoma-derived CAOV-3 cells express ADORA2A transcript, and its activation reduce cell migration. (A) ADORA2B transcript was amplified by RT-PCR from cDNA synthetized from total RNA of CAOV-3 cells; SOD2 transcript was amplified as constitutive control, amplicon was sequenced and analyzed in BLAST platform, confirming its identity. (B) CAOV-3 cells were stimulated with BAY-606583 (0.1, 1 and 10 mM) by 16 h, and cell migration was evaluated by scratch assay; representative pictures are shown in (C). *$p<0.05$; n=4 experiments done by triplicate.
